# Medical advice for sick-reported students in a Dutch vocational school: a process evaluation

**DOI:** 10.1093/heapro/daad019

**Published:** 2023-03-22

**Authors:** Kristel Jenniskens, Jessie Jacoba Maria Meis, G A Rixt Zijlstra

**Affiliations:** Faculty of Health, Medicine and Life Sciences, Maastricht University, P.O. Box 616, 6200 MD, Maastricht, The Netherlands; Department of Knowledge and Innovation, Public Health Services South Limburg (GGD Zuid Limburg), P.O. Box 33, 6400 AA, Heerlen, The Netherlands; Department of Health Services Research, Care and Public Health Research Institute, Maastricht University, P.O. Box 616, 6200 MD, Maastricht, The Netherlands; Department of Health Policy and Research, Public Health Services Flevoland (GGD Flevoland), P.O. Box 1120, 8200 BC Lelystad, The Netherlands

**Keywords:** process evaluation, vocational education, medical absence, intervention

## Abstract

Medical Advice for Sick-reported Students (MASS) is an intervention that aims to reduce medical absenteeism and prevent dropout among students. The current study reports on a process evaluation of the implementation of MASS at a vocational school in the Netherlands. The evaluation included the implementation process, fidelity, context, and participant satisfaction. The study had a qualitative case study design. Data was gathered through semi-structured interviews with relevant stakeholders, including a child and youth healthcare physician, MASS coordinators, career advisors, mentors, and students with concerning sickness absence. MASS was largely implemented as intended, but some deviations from the original intervention were found. For example, not all mentors identified concerning sickness absence through recommended criteria. A fit between the intervention and the values of the involved organizations was found. Facilitating contextual factors were identified, such as a perceived need for reducing school absence recognized within the care network, as well as hampering contextual factors, for example the limited visibility of students’ absence during the COVID-19 pandemic. Participants were generally satisfied with MASS and its implementation. Overall, MASS was implemented well according to interviewees, but several improvement points for both the implementation and execution of MASS were identified. These include full implementation across the setting, providing and repeating necessary trainings, minimizing administrative burden, and securing financial and human resources for sustainment of the intervention. These points could help to guide future implementation efforts, as they may help to overcome common barriers to implementation.

## INTRODUCTION

School absenteeism is a major problem due to its link with increased dropout rates (Cabus and De Witte, 2015; Theunissen *et al.*, 2015). In 2021, nearly 10% of the people in the European Union aged 18–24 years who finished secondary education did not attend further education (Eurostat, 2022). Furthermore, school absenteeism and dropout are associated with negative health outcomes such as increased mortality, health risk behaviour and lower health related quality of life (Eaton *et al.*, 2008; Lawrence *et al.*, 2016; van den Toren *et al.*, 2019). Because of these associations, school absenteeism can be regarded as a public health problem, yet around the world little research has been conducted into interventions tackling this problem.

In West Brabant, the Netherlands, however, an intervention called Medical Advising Sick-reported Students (MASS) has been developed by the preventive Child and Youth Healthcare department of the Public Health Services (GGD). MASS is aimed at reducing medical absenteeism and preventing dropout among students (Nederlands Centrum Jeugdgezondheid, 2020). The intervention operates at a school- and individual-level (Nederlands Centrum Jeugdgezondheid, 2020). At the school level, medical absenteeism policy is optimized and criteria to detect students with concerning sickness absence are integrated into the school’s policy (Vanneste, 2014). On the individual level, students with concerning sickness absence are identified and supported by the school and, if needed, referred to a child and youth healthcare (CYH) physician from the GGD to assess their capability to attend school (Vanneste, 2014).

MASS has previously been shown effective in reducing sickness absence and dropout among secondary school students (Vanneste *et al.*, 2012, 2016) and vocational school students (Van der Vlis *et al.*, 2017; van den Toren *et al.*, 2020). Additionally, a process evaluation of how MASS was implemented at eight schools showed that in most cases, the intervention was delivered as intended, that CYH physicians considered MASS to be useful, and that most students were satisfied with MASS (van den Toren *et al.*, 2020). Despite these promising findings, the evaluation did not include other stakeholders, such as school personnel, and contextual factors were not assessed.

In the past years, MASS has been implemented across The Netherlands in both secondary schools and vocational schools (Nederlands Centrum Jeugdgezondheid, 2020). After a pilot implementation of MASS in one educational program in August 2020, the program was disseminated to all first year students at one location of a vocational school in South Limburg, the Netherlands (hereafter ‘the vocational school’) in August 2021. The intention was to gradually disseminate MASS further across the school over the coming years if it proved to be acceptable, feasible, and effective. Alongside the implementation of MASS at this vocational school, a process evaluation was performed. According to Saunders *et al.* (2005), a process evaluation can provide insight into how a program was implemented and how the implementation might have affected the impact or outcomes of the program. The current study reports on the findings of a process evaluation of MASS and seeks to answer the following research questions:

- What were the steps in the implementation process?
- To what extent was MASS implemented as intended?
- What contextual factors affected the implementation?
- What were the experiences of the various stakeholders in the MASS intervention at the vocational school?

### Theoretical framework

Two frameworks offering guidance in process evaluations are the Medical Research Council (MRC) guidance for evaluating complex interventions from Moore et al. (Moore *et al.*, 2015) and the Process Evaluation Plan (PE Plan) from Saunders et al. (Saunders *et al.*, 2005). According to the MRC guidance, an intervention’s outcomes are the combined result of the intervention, its implementation, its mechanisms of impact and the context in which it was implemented (Moore *et al.*, 2015). Additionally, the context influences the implementation. According to the PE plan, important elements of implementation to consider in a process evaluation include: implementation process, fidelity, dose delivered, exposure, participant satisfaction, adaptations, reach and recruitment (Saunders *et al.*, 2005).

**Fig. 1:**
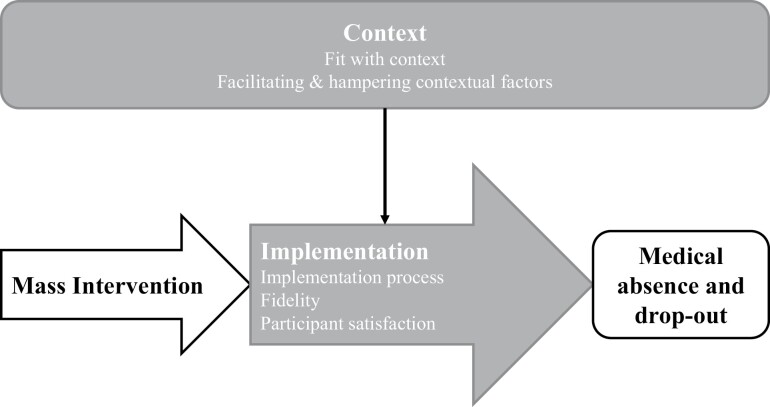
Conceptual framework for the evaluation of the implementation of MASS.

Aspects of both frameworks were combined into a conceptual framework for the current study (Figure 1). As shown in this framework, the process evaluation focussed on the implementation of MASS at the vocational school in terms of the implementation process, fidelity, and participant satisfaction, and on the context in which MASS was implemented in terms of the fit between MASS and its context, and contextual factors that facilitated or hampered the implementation.

## METHODS

A qualitative case study design was chosen. It is suitable for a process evaluation, because process evaluations describe the implementation of an intervention into a specific context, for which the experiences and perspectives from different stakeholders are required (Saunders *et al.*, 2005). A case study is suitable for a process evaluation, because it ‘investigates a contemporary phenomenon in depth and within its real-life context’ and ‘relies on multiple sources of evidence’ (Yin, 2009). This study was reported following the Standards for Reporting Qualitative Research (O’Brien *et al.*, 2014). All interviewees signed an informed consent form before participation.

### Participants and setting

The setting is a vocational school in South Limburg, the Netherlands. Dutch vocational school follows pre-vocational training in secondary school. There are four levels: (i) assistant training, preparing students for an assistant job or further education, (ii) basic vocational training, preparing students for executive jobs, (iii) professional training, preparing students for more autonomous functions, (iv) middle-management training, preparing students for fully autonomous functions (Rijksoverheid, 2023b). Furthermore, gaining practical experience through internships or apprenticeships are an important part of all vocational programs (Rijksoverheid, 2023a [in Dutch only]). The vocational school in the current study offers education on all four levels, and in various fields, such as health care, food, entertainment, or ICT. The school has locations in three cities, MASS was implemented in one city. In 2019, most vocational school students were between 18 and 22 years old (MBO Raad, 2020).

MASS was implemented in collaboration with the CYH department from the GGD South Limburg, a public health institute. Dutch CYH follows the growth and development of children between 0 and 18 years old (GGD Zuid Limburg, 2023). Sampling was purposive to include stakeholders in the implementation that could provide in-depth information on their experiences (Moser and Korstjens, 2018). Four types of stakeholders from both organizations were included. First, both coordinators of MASS at the vocational school and the GGD were included. Second, all twelve career advisors from the school were invited, because they trained and supported mentors in applying MASS. Third, those carrying out the MASS intervention in practice were invited for interview: the mentors and the CYH physician who was involved in MASS at the vocational school. All 136 mentors of first year classes received an invitation for interview at the end of a questionnaire that was part of another study. Additionally, the CYH physician and a career advisor randomly selected mentors. Last, the target population of MASS was included, that is, students with concerning sickness absence. Seventeen students who had seen the CYH physician for concerning sickness absence between January and May 2022 were invited for interview. Additionally, mentors who had participated in an interview were asked to select students with concerning sickness absence that had not seen the CYH physician. Career advisors, mentors and students were recruited until data saturation was reached. The criteria for data saturation were: (i) a minimum of four interviews per stakeholder group, (ii) the last two interviews did not lead to new information, and (iii) two researchers (KJ and JM) agreed that data saturation was reached.

### Intervention description

In MASS, students who report sick are guided through five steps, described in the MASS handbook for vocational schools from (Vanneste, 2014). An important basis for MASS is to approach sickness absence from a caring perspective, instead of a controlling perspective (Vanneste, 2014). First, the mentor contacts students about their absence and monitors the absence (step 1). Once the absence meets the MASS criteria, the mentor invites students for a meeting about their absence (step 2). The MASS criteria are determined by the school, but the handbook suggests at least seven consecutive days of sickness absence, or four or more sick reports in three months. If mentors need assistance in estimating a student’s capability to attend school, they can refer them to a CYH physician (step 3). When doing so, the mentor should inform the student about the purpose of this referral. In step 4, the student has a consultation with a CYH physician, who assesses their capability to attend school, assists them in formulating a reintegration plan for returning to school, and can refer the student to specialized care. The reintegration plan describes concrete steps for returning to school (Vanneste, 2014). In step 5, the school monitors the student’s absence and adherence to the action plan.

### Data collection

Data was collected through individual interviews with stakeholders between May 4th and June 10th, 2022. All interviews were in Dutch, and were conducted by KJ who at the time was a Master’s student from Maastricht University following the Master Healthcare Policy, Innovation and Management, and an intern at the GGD South Limburg. She had experience in conducting and analysing semi-structured interviews.

All interviews were semi-structured, following a topic lists drafted beforehand. All interviews started with a general introduction in which the interview’s purpose and anonymity were explained, and permission for recording was asked. Prior to the interviews, the researcher met the CYH physician, coordinators, and some of the career advisors, because they worked in the same office and helped to approach other stakeholders. Because of this, the researcher had pre-knowledge about the implementation process before the start of the interviews. To minimize steering during the interviews towards this pre-knowledge, the topic lists did not include specific questions about this. However, when needed, the interviewer did use examples during the interview that were based on this pre-knowledge. The researcher had not met the mentors and students prior to the interviews. Researcher’s characteristics did not influence the research questions, approach, methods, results, and transferability.

The interviews with the coordinators, career advisors, mentors and CYH physician covered four topics. First, the implementation process was discussed through questions such as: what steps were taken to implement MASS? The second topic addressed fidelity, for example: to what extent was MASS implemented as intended? It also included questions about the execution of MASS, for example: how was concerning sickness absence identified? Third, the context was discussed, for example: how does MASS fit with the vocational school? Lastly, participants were asked about their experiences with MASS, for example: what is your opinion about MASS?

Interviews with the students covered three topics. First, students were asked if they had previously heard of MASS. Next, their experiences with different parts of the intervention were discussed, through questions such as: what did you think about the consultation with the CYH physician? Lastly, students were asked about the steps taken after their consultation, for example: what agreements did you make with the CYH physician considering reintegration?

### Data analysis

The first step in the data analysis was the verbatim transcription in Dutch of recorded interviews in Word by the interviewer, which was anonymized. Next, transcripts were analysed in Dutch by KJ through deductive coding based on the conceptual model (Figure 1), including: ‘implementation process’, ‘fidelity’, ‘participant satisfaction’, ‘contextual factors’, and ‘fit with context’. Data that did not fit these codes were inductively coded through open coding, after which open coded data was reassigned into more abstract conceptual categories through axial coding (Scott and Medaugh, 2017). Data was coded by the researcher in NVivo version 12. Quotes selected for the result section were translated to English after the coding process.

### Trustworthiness

Several strategies were used to enhance trustworthiness. First, participants were send a summary of the interview, to member-check the interpretations of the researcher, and enhance credibility (Korstjens and Moser, 2018). Second, multiple stakeholders were interviewed as a means of data triangulation, through which credibility and transferability were increased (Korstjens and Moser, 2018). Transferability was increased through sampling until data saturation (Korstjens and Moser, 2018).

## RESULTS

In total, 20 participants were interviewed, including: one CYH physician, two coordinators, five career advisors, six students, and six mentors (of which three worked with MASS and three had not been introduced to MASS yet). The mean age of the students was 19.2 years (SD = 2.5, range 17–24), five were female and one was male. All but one student were referred to the CYH physician.

### Implementation process

Most interviewed professionals indicated that the decision to implement MASS was based on a need for preventive action against sickness absence among vocational school students.

*“[One CYH physician] mainly saw multi-problem cases with prolonged school absence. And we said: we should act a lot earlier, and that is why MASS was brought to life”*—coordinator GGD.

The coordinator from the school explained that the implementation of MASS was planned, and started with an information session for all career advisors. This session was organized by the CYH physician and a career advisor, who had been involved in a previous pilot. During the session, the method of MASS and the implementation plan were presented. Next, career advisors were asked to inform the mentors of first year students within their educational departments.

*[Career advisors] have good connections within the educational departments … we were not going to drop it, top-down, but implement it through these relationships.*

—coordinator school.

Career advisors received a PowerPoint presentation on MASS from the coordinator, which they could use to inform their educational departments about MASS. No further training activities were organized for the implementation of MASS.

The coordinator and several career advisors mentioned that MASS was not introduced in all educational departments in the school year 2021/2022. Several explanations were given, that is, MASS was not a priority for everyone, due to relatively low absence numbers in some departments or other circumstances requiring more attention, and a lack of incentive for career advisors to introduce MASS to their educational department.

*“It is to some extent non-committal for career advisors [to introduce MASS], we are not charged for not introducing it”*—career advisor.

### Fidelity

MASS was generally implemented as intended, but some deviations were found as well. On the school level, both coordinators indicated that MASS had not been fully added to the school’s absence policy, because it had not yet been implemented at all locations of the school.

*“We have one absence policy … which applies to all locations … [adding MASS to this policy] would mean we have to be able to consult the CYH physician at all locations, which is not the case”*—coordinator school.

On the individual level, all mentors who work with MASS and all students stated there was immediate contact between the mentor and student after the first sick report (step 1). Of the three mentors that were not yet introduced to MASS, two indicated they also directly contact a student after their first sick report.

Regarding the identification of concerning sickness absence (step 2), the coordinator of the GGD and the CYH physician suspected that the MASS criteria were not properly used, since only students with complex problems were referred.

*“[Students] should be identified earlier, and the [MASS] criteria should be used more”*—CYH physician.

Only one career advisor reported applying the MASS criteria. Others knew the criteria, but did not apply them. Instead, they based their concerns on intuition and a student’s history. All mentors who worked with MASS explained they planned an absence meeting when a student’s sickness absence became concerning. Sometimes the career advisors joined these meetings.

*“For cases that need extra care or extra attention, mentors can involve me”*—career advisor.

According to all mentors who work with MASS, parents were present during the absence meeting if a student was younger than 18 years or upon the student’s request if the student was older than 18 years. All students indicated they had an absence meeting at some point. Mentors who worked with MASS agreed that the goal of the meeting is to discuss the student’s situation and the need for further support. Mentors who were not yet introduced to MASS, also mentioned they had absence meetings with students when their sickness absence was concerning, which they based on intuition or patterns in absence.

Regarding the referral to the CYH physician (step 3), all career advisors and mentors agreed they could easily contact the CYH physician. The CYH physician was generally satisfied with how students were referred, although sometimes students were not informed by school about the purpose of the referral.

*“If [students are not informed about the purpose of the consultation], and that occurs, a student does not really know what they come in for”*—CYH physician.

According to the mentors and career advisors, the purpose for referral was explained and students knew the purpose prior to their consultation with the CYH physician. All mentors that worked with MASS had referred students to the CYH physician in the past school year. Yet, some career advisors had not yet referred students to the CYH physician. They indicated that prior to referring to the CYH physician, they referred students to their general practitioner, or psychologist if a student was already undergoing treatment.

*“I think the CYH physician is called upon if we could not get in touch [with the general practitioner or another caregiver]”*—career advisor.

Mentors who were not yet introduced to MASS never referred students to the CYH physician, but did sometimes ask students to discuss their capability to attend school with other caregivers, such as a treating psychologist.

Regarding the consultation with the CYH physician (step 4), both the CYH physician and students explained they discussed the students’ situation and drafted a reintegration plan together. Furthermore, the CYH physician and some students mentioned that the CYH physician sometimes referred students to other professionals, such as psychologists. After a consultation, the CYH physician stated they reported to the mentor, but only if the student agreed. Career advisors and mentors also stated they usually received feedback from the CYH physician. This feedback mostly included information about the student’s capability to attend school and the reintegration plan.

*“The feedback I received was that this student really was not able to attend school at this point”*—mentor.

As part of step 5, mentors who had referred students to the CYH physician, all monitored their absence and adherence to the reintegration plan after the consultation. Students confirmed they had regular contact with their mentor after seeing the CYH physician. For mentors to whom MASS was not yet introduced, it is unclear to what extent they monitor sickness absence.

### Context

MASS fitted the context of both the GGD and the vocational school. The CYH physician and coordinator of the GGD mentioned that as a preventive intervention, MASS fitted the GGD’s focus on public health. However, the CYH physician also mentioned that their work at the vocational school resembled that of an occupational physician, more than that of a CYH physician, because they developed reintegration plans and often worked with young adults.

*“It doesn’t fit completely [with my job], because I see a lot of young adults [who are older than 18]”*—CYH physician.

Considering the vocational school, career advisors and mentors agreed that MASS resembled the school’s previous strategy to tackle sickness absence. Nevertheless, MASS helped them to be more aware of sickness absence and to involve the CYH physician if needed.

*“For me it quickly became clear that we already take the right steps … although I think that if I consult the criteria, it might be easier to make decisions … For me it’s also the first time I consulted a CYH physician”*—mentor.

Several facilitating contextual factors were found. To begin with, absence among vocational students was high on the agenda. The coordinator of the GGD mentioned that MASS was part of a broader action plan for reducing school absence of local governments, the GGD, the bureau for Early School Leaving (Dutch abbreviation: VSV), and vocational schools. Furthermore, all mentors agreed that reducing absence was high on the school’s agenda.

*“More and more resources are spent by [the school] to reduce absence”*—mentor.

Also, career advisors and mentors mentioned that prior to the implementation of MASS there was a need to consult an external party for cases of concerning sickness absence.

*“We had a need for MASS, because we always struggle with: how do we handle long term medical sickness absence?”*—career advisor.

Moreover, the CYH physician explained that with MASS they could plan consults up to an hour. An hour allows them to discuss a student’s situation extensively, and to assess the student’s needs and develop a reintegration plan together.

*“A general practitioner could also do this … but they don’t have the time. I can really take the time”*—CYH physician.

Finally, according to the coordinator of the GGD, tenacity of the GGD has helped to prevent delay in the implementation of MASS.

*“Even if finances are not secured yet, we often do continue”*—coordinator GGD.

Hampering contextual factors were identified as well. First, the CYH physician and a mentor described how the COVID-19 pandemic made it more difficult to identify students with concerning sickness absence.

*“Students and sickness absence were less visible [during lockdowns]”*—CYH physician.

Second, according to the CYH physician, students older than 18 years had to be referred to adult mental healthcare, which was new for the CYH physician.

*“I am not familiar with this social map [of adult mental healthcare] … I cannot directly refer to adult mental healthcare, like for youth mental healthcare”*—CYH physician.

Lastly, the coordinator explained how her switch to a different department than CYH within the GGD, while remaining coordinator of MASS, was a barrier.

*“I remained coordinator, while MASS is a part of CYH. You notice that therefore, CYH insufficiently adopts MASS [for vocational schools]. We should have a coordinator from CYH.”*—coordinator GGD.

### Participant satisfaction

All interviewed professionals were mostly satisfied with the implementation of MASS. Generally, the implementation through the relationships between career advisors and mentors was appreciated.

*“We already know the mentors, so yes, I support [this method]”*—career advisor.

All career advisors were satisfied with the presentation they received about MASS. Afterwards, they all felt competent to present MASS to their educational departments. All mentors who attended the presentation indicated that the steps in MASS were clear and they felt competent to apply MASS. Furthermore, interviewees were generally satisfied with the communication between the parties involved in the implementation.

Interviewees also experienced some challenges. First, the coordinator of the GGD stated that the municipality’s policy officers of public health were not involved in the initial deliberations about MASS causing concerns about the available financial budget.

*“Everything occurred outside the field of view of the policy officers of public health. So once the budget had to be approved, they were insufficiently informed”*—coordinator GGD.

Second, although trained in MASS for secondary schools, the CYH physician missed a training in reintegration and assessing a student’s capability to attend school, and currently experienced insufficient expertise to deal with this during consultations. Third, one mentor who worked with MASS highlighted that referring a student to the CYH physician was an administrative burden.

*“You have to look everything up, gather all the information you need... All of that together, makes it a lot of work”*—mentor.

All professionals who worked with MASS deemed MASS a necessary and useful intervention for addressing sickness absence and preventing dropout. Career advisors and mentors who worked with MASS particularly appreciated having an external party available to recommend on a student’s maximum workload given their medical conditions. All mentors who did not yet work with MASS also expected the intervention to be supportive. Some career advisors appreciated that MASS allowed them to approach sickness absence from a caring instead of a controlling perspective.

*“Not with an accusing finger like you have to go to school, but more like what’s going on, and can we help?”*—career advisor.

Students’ experiences with the support they received from school were mixed. Half of them were satisfied, and felt supported throughout their absence.

*“[My mentor] was very involved from day one; she came by, and clearly explained what I could do. I was very satisfied with her response”*—student.

The other students felt supported in the end, but experienced some difficulties when their sickness absence started. Two students felt not taken seriously by their mentor until their parents got involved and another student felt that the mentor should have referred to the CYH physician earlier.

*“Maybe if I had gone earlier [to the CYH physician], things would have been different”*—student.

All students who visited the CYH physician were satisfied with the physician’s support. Two students appreciated that after the appointment, they were referred to the care they needed.

*“Since [the appointment] I felt that something started to get going, because he could refer me to a psychologist”*—student.

Students had mixed needs considering parental involvement by school. Some students were satisfied about their parents not being involved, while another was satisfied with the involvement of a parent and another student indicated missing the involvement of a parent.

*“My mentor has barely to no contact with parents. I would want this to be more, I think it is important”*—student.

Most students felt that MASS had helped them. Some indicated the referral to specialized care helped, while others indicated they benefited from the reintegration plan. Career advisors and mentors found it difficult to recognize immediate results from MASS, but some noticed improved contact between the student and school and a better understanding of the student’s situation.

### Future of MASS

All professionals who already worked with MASS preferred to continue doing so. Nevertheless, mentors who worked with MASS indicated the need to repeat the instruction next school year. All mentors who did not yet work with MASS indicated they were willing to start working with the intervention, provided they will receive instructions. Furthermore, one career advisor and some mentors preferred the referral to the CYH physician to be performed by the career advisors. Career advisors are familiar with this procedure and know which information the CYH physician requires. Additionally, this would relieve the mentors’ workload.

*“You have 25 students. If five or six students are not doing well, you really have to plan. It can be a lot.”*—mentor.

To continue working with MASS at the vocational school will require dissemination to all locations and ensuring continuation. Both coordinators stated that finances are currently only secured until the school year 2022/2023, and that currently only one CYH physician is available to work with MASS in vocational education in the region South Limburg This makes the future of MASS at the vocational school unsure.

## DISCUSSION

This qualitative study reported on the outcomes of a process evaluation of the implementation of the MASS intervention, aimed to reduce medical absenteeism and prevent dropout among students, at a vocational school. The process evaluation focussed on the implementation process, fidelity, context, and participant satisfaction. Concerning the implementation process, MASS was implemented by first informing the career advisors, who then presented MASS to the first year mentors of their educational departments. No training activities were organized for those who worked with MASS. The five steps of MASS described in the handbook from Vanneste (Vanneste, 2014) were recognized in practice and the qualitative results showed that MASS was largely implemented as intended. Overall, MASS fitted well within the context of the GGD and the vocational school. Various contextual factors facilitated and hampered the implementation of MASS, for example a perceived need for reducing school absence among vocational students recognized within the care network and the limited visibility of students’ absence during the COVID-19 pandemic. Participant satisfaction with both the implementation and MASS were generally high.

The findings indicated that the implementation of MASS at the vocational school followed stepwise plan, which resembled recommended implementation plans from the Dutch Centre for Youth Health (NCJ) (NCJ, 2022) and from Parker et al. (Parker *et al.*, 2021). However, barriers, facilitators, and readiness to change among stakeholders were not assessed prior to the implementation of MASS at the vocational school, as is suggested in several approaches to developing implementation plans (Grol *et al.*, 2013; Fernandez *et al.*, 2019; Parker *et al.*, 2021). The implementation might have benefited from such an assessment. For example, MASS was only partially implemented, that is, not all educational departments were introduced to MASS. The identification of barriers prior to the implementation might have helped to anticipate this and include strategies in the implementation plan to prevent partial implementation. Since a study by Østensen et al. (Østensen *et al.*, 2020) showed that partial implementation of an intervention was associated with limited outcomes compared to full implementation, the analysis of barriers and facilitators prior to implementation MASS could thus also help to prevent limited effectiveness of the intervention. Following a structured approach to develop an implementation plan might therefore help to consider such an analysis and consequently improve the implementation process.

Another factor related to the implementation of MASS that might have an impact on the intervention’s outcomes at the vocational school is fidelity. Although interviewees indicated that MASS was largely implemented as intended, some parts of the intervention were not executed sufficiently. This is an important finding, as Clarke et al. (Clarke *et al.*, 2014) and Durlak and DuPre (Durlak and DuPre, 2008) found that high fidelity is associated with better intervention outcomes. As such, the finding in the current study that one student thought they were referred to the CYH physician too late might be explained by the insufficient usage of the MASS criteria for identifying concerning sickness absence. Another deviation from the MASS protocol was that students were not always informed beforehand about the purpose of the referral to the CYH physician. This is an important finding, because in an evaluation of MASS from Schmits et al. (Schmits *et al.*, 2021) students reported they appreciated an explanation from their school about the purpose of a referral to the CYH physician. Finally, the finding that some students did not feel taken seriously by school in light of their sickness absence may imply that not all mentors currently approached sickness absence from a caring perspective, as is suggested by Vanneste (Vanneste, 2014). These deviations from the MASS handbook emphasize the importance of training mentors in having consultations with students as recommended by the MASS handbook (Vanneste, 2014), and repeating instructions about MASS, which was acknowledged by the mentors.

One important hampering contextual factor was the role of the CYH physician in MASS for vocational schools resembling that of an occupational physician. This could be explained by the fact that internships or apprenticeships are an important part of vocational programs (Rijksoverheid, 2023a [in Dutch only]). As a result, the CYH physician who followed a training for MASS in secondary school now got an additional task of assessing students’ ability to reintegrate into their internship or apprenticeship. This is not considered to be a task of Dutch CYH physicians according to their professionals standard (AJN Jeugdartsen Nederland, 2021). This might also explain why the CYH physician indicated to feel unsure about reintegration and assessing students’ ability to attend school although they did not specifically mention reintegration into internships and apprenticeships as a difficulty. Still, the results of the current study stress the need for additional training for CYH physicians who have a role in MASS at vocational schools.

Another important hampering contextual factor was the high workload experienced by mentors. Combined with the perceived high administrative burden of referring students to the CYH physician, this could lead to resistance among mentors to use MASS. This assumption is supported by systematic reviews from Liu et al. (Liu *et al.*, 2019), Tancred et al. (Tancred *et al.*, 2018), and Wierenga et al. (Wierenga *et al.*, 2013), which identified high work demands as a barrier to the implementation of health interventions. A possible solution is to appoint the responsibility of referring students to the CYH physician to career advisors to minimize the administrative pressure for mentors, which some participants in the current study preferred.

### Strengths and limitations

Strengths of this study were enhanced credibility and transferability of the results through member-check, data triangulation, and inclusion of stakeholders until data saturation where possible. Furthermore, the study population included key stakeholders in the intervention and its implementation, increasing the likelihood that all relevant experiences were collected. Another strength of this study is the systematic approach to conducting and designing the process evaluation, for which it followed Moore *et al.* (Moore *et al.*, 2015) and Saunders *et al.* (Saunders *et al.*, 2005). Furthermore, the qualitative nature of this process evaluation helped gain in-depth insights into the experiences of various stakeholders with MASS and its implementation.

A limitation was the potential risk for translation bias, as all interviews and transcripts were in Dutch, while the report was in English. Furthermore, the transcripts were analysed by only one researcher (KJ). However, prior to the coding process, the code tree was established in accordance with a second researcher (JM). Another limitation was that the students and career advisors were not randomly selected. As is common in qualitative research, it is possible that only participants with strong opinions responded, which may have led to selection bias. Selection bias among the career advisors was limited as much as possible by also asking career advisors how they thought other career advisors would answer the interview questions. Lastly, for some stakeholder groups data saturation proved impossible, due to the limited numbers employed and involved in MASS. Potentially this may limit the transferability of data.

### Recommendations

Based on the findings of this process evaluation, several recommendations for practice were composed. First, full implementation of MASS is recommended so that all students receive the same care. Second, executing stakeholders should be provided with necessary trainings, to enhance their competence in MASS. Third, the instruction of MASS should be repeated in all educational departments, with special attention for the MASS criteria, the importance of explaining to students the purpose of a referral to the CYH physician beforehand, and the importance of taking on a caring, instead of controlling attitude towards students. Fourth, administrative pressure should be minimized as much as possible, for example by delegating certain responsibilities to other parties. Last, securement of sustainable finances and personnel are required to secure MASS at the vocational school. These recommendations might also help other future implementation efforts. For example, it is likely that the implementation of most interventions could benefit from providing necessary trainings and repeating instructions regularly, because this ensures that end-users are qualified and skilled to work with the intervention.

Several recommendations for research were identified as well. To begin with, a structured approach to developing an implementation plan is recommended to enhance the implementation success. Next, more process evaluations for MASS at different locations are needed to increase transferability of data. Lastly, regular process evaluations are recommended for any public health intervention, as it can help to explain intervention outcomes and to identify improvement points for future use of the intervention.

## CONCLUSION

The aim of this process evaluation was to identify improvement points for the implementation and execution of MASS at a vocational school in South Limburg, the Netherlands. Findings showed that the implementation of MASS in the past year went well, but some improvement points were identified, for example, full implementation of MASS and providing necessary trainings for executing stakeholders. However, the future of MASS at the vocational school is unsure, because sustainable financing is not yet secured and there are currently not enough CYH physicians to disseminate MASS to other locations of the school. As the users and target population of the intervention were satisfied with MASS, it is recommended to implement MASS for vocational students. Monitoring of the effects of MASS in practice, including an evaluation of cost-effectiveness, should be part of future research.

## Supplementary Material

daad019_suppl_Supplementary_Appendix
