# Supplementary material for: Medical advice for sick-reported students in a Dutch vocational school: a process evaluation
Source: Health Promot Int. 2023 Mar 22;38(2):daad019. doi: 10.1093/heapro/daad019 (PMC10472874; doi:10.1093/heapro/daad019)
Supplement: daad019_suppl_Supplementary_Appendix [file daad019_suppl_supplementary_appendix.docx]

## Appendix 1: interview topic lists

**Project leaders**

| ***Introduction:***   - Study- and interview purpose - Anonymity, signing informed consent form - Permission for recording | |
| --- | --- |
| ***Topic*** | ***Questions*** |
| **Introduction interviewee** | Could please introduce yourself?   - Work experience - Role is MASS |
| **Implementation process** | How did the idea to implement MASS come about?   - People involved - Resistance against idea   Which steps were taken to implement MASS?   - Materials used   - Who provided/developed them?   - Clarity, usefulness, completeness - Training- and supporting activities   - Who provided these?   - Clarity, usefulness, completeness   Who were involved in the implementation of MASS?   - Communication |
| **Fidelity and adaptations** | To what extent was MASS implemented as intended?  What adaptations were made to the original intervention?   - Why? |
| **Context** | Which characteristics from your organisation facilitated the implementation of MASS (and how)?  Which characteristics from your organisation hindered the implementation of MASS (and how)?  To what extent does MASS fit with your organisation?   - Norms and values of organisation - Difference from previous approach to medical absence in vocational students |
| **Satisfaction** | How do you rate MASS?  How do you rate the implementation of MASS?  Are you willing to keep working with MASS in the future? |
| ***Closing***   - Is there anything I missed that I should know? - Questions interviewee - Explain summary of interview + ask for feedback - Thanks | |

**CYH physician**

| ***Introduction:***   - Study- and interview purpose - Anonymity, signing informed consent form - Permission for recording | |
| --- | --- |
| ***Topic*** | ***Questions*** |
| **Introduction interviewee** | Could please introduce yourself?   - Work experience - Role is MASS |
| **Implementation process** | How did the idea to implement MASS come about?   - People involved - Resistance against idea   Which steps were taken to implement MASS?   - Materials used   - Who provided/developed them?   - Clarity, usefulness, completeness - Training- and supporting activities   - Who provided these?   - Clarity, usefulness, completeness   Who were involved in the implementation of MASS?   - Own involvement - Communication |
| **Fidelity and adaptations** | To what extent was MASS implemented as intended?  What adaptations were made to the original intervention?   - Why? |
| **Execution of MASS** | How were students with concerning medical absence identified?   - How were they referred to you”? - Satisfaction with referral process   How did the consultation with students with concerning medical absence go?   - Development of action plan - Presence of parents - Difficulties   - What would help to overcome these in the future? - Involvement after consultation   To what extent was the procedure of MASS clear to you? |
| **Context** | Which characteristics from your organisation facilitated the implementation of MASS (and how)?  Which characteristics from your organisation hindered the implementation of MASS (and how)?  To what extent does MASS fit with your organisation and job?   - Norms and values of organisation - Difference from previous approach to medical absence in vocational students |
| **Satisfaction** | How do you rate MASS?  How do you rate the implementation of MASS?  Are you willing to keep working with MASS in the future? |
| ***Closing***   - Is there anything I missed that I should know? - Questions interviewee - Explain summary of interview + ask for feedback - Thanks | |

**Career advisors**

| ***Introduction:***   - Study- and interview purpose - Anonymity, signing informed consent form - Permission for recording | |
| --- | --- |
| ***Topic*** | ***Questions*** |
| **Introduction interviewee** | Could please introduce yourself?   - Work experience - Role is MASS |
| **Implementation process** | To what extent were you involved in the implementation of MASS? *If involved, follow up questions:*   - Which steps were taken to implement MASS?   - Materials used     - Who provided/developed them?     - Clarity, usefulness, completeness   - Training- and supporting activities     - Who provided these?     - Clarity, usefulness, completeness   Who were involved in the implementation of MASS?   - Own involvement - Communication |
| **Fidelity and adaptations** | To what extent was MASS implemented as intended?  What adaptations were made to the original intervention?   - Why? |
| **Execution of MASS** | How were students with concerning medical absence identified?   - By whom? - Own involvement - Correctness of method   What happened after a student with concerning medical absence was identified?   - Taken by whom? - Own involvement - Correctness of method   Have you yourself referred any students to the CYH physician for their medical absence?   - How do you determine whether or not to refer? - Self-efficacy to decide whether or not referral is necessary - Referral process   - Satisfaction - What did the CYH physician report back to you after the referral?   - Satisfaction   To what extent do you monitor presence and the action plan after a student went to the CYH physician? |
| **Context** | Which characteristics from your organisation facilitated the implementation of MASS (and how)?  Which characteristics from your organisation hindered the implementation of MASS (and how)?  To what extent does MASS fit with your organisation and job?   - Norms and values of organisation - Difference from previous approach to medical absence in vocational students |
| **Satisfaction** | How do you rate MASS?  How do you rate the implementation of MASS?  Are you willing to keep working with MASS in the future? |
| ***Closing***   - Is there anything I missed that I should know? - Questions interviewee - Explain summary of interview + ask for feedback - Thanks | |

**Mentors**

| ***Introduction:***   - Study- and interview purpose - Anonymity, signing informed consent form - Permission for recording | |
| --- | --- |
| ***Topic*** | ***Questions*** |
| **Introduction interviewee** | Could please introduce yourself?   - Work experience - Role is MASS   Are you familiar with MASS?   - If yes: were there any students with concerning medical absence in your class this year?   - If yes: part a questions   - If no: part b questions - If no: *short explanation of MASS*, does this sound familiar?   - If yes: were there any students with concerning medical absence in your class this year?     - If yes: part a questions     - If no: part b questions   - If no: part c questions |
| **Implementation process** | *Part a and b:*  When did you first hear about MASS?   - How, what information - Which steps were taken to implement MASS to your knowledge?   What materials did you receive to assist you in working with MASS?   - From whom - Clarity, usefulness, completeness   What training- and supporting activities were organised for you?   - By whom - Clarity, usefulness, completeness   *Part c:*  Did you ever follow a training about medical absence in students?   - If yes: organised by whom, clarity, usefulness, completeness - If no: would you be willing to follow such a training a why?   - What topics do you think such a training should cover? |
| **Execution of MASS** | *Part a, b, and c:*  What do you do when a student reports sick?  What do you do when the medical absence of a student concerns you?   - *Part a and b:* Knowledge of MASS criteria   - Application of MASS criteria - Sense of responsibility - To what extent are you actively identifying concerning medical absence in your students on a day to day basis?   How did conversations with students about their medical absence go?   - Self-efficacy - Parental involvement - Difficulties   - What would help to overcome these?   *Part a and b:*  How do you decide whether or not to refer a student to the CYH physician?   - Referral process   - Satisfaction   *Part a:*  What does the CYH physician report back to you after a consultation with a student?   - Satisfaction   To what extent do you monitor presence and the action plan after a student went to the CYH physician?   - Self-efficacy   *Part c:*  Do you refer students to other professionals when you think their medical absence is concerning?   - To whom? - Did you ever consider referring to a CYH physician? |
| **Context** | *Part a and b:*  Which characteristics from your organisation facilitated the implementation of MASS (and how)?  Which characteristics from your organisation hindered the implementation of MASS (and how)?  To what extent does MASS fit with your organisation and job?   - Norms and values of organisation - Difference from previous approach to medical absence in vocational students   *Part c:*  *short explanation of MASS* upon hearing this, how do you think MASS relates to your tasks as a mentor?   - Fit with job - Fit with organisation   To what extent does MASS differ from your current approach to concerning medical absence in students? |
| **Satisfaction** | *Part a and b:*  How do you rate MASS?  How do you rate the implementation of MASS?  Are you willing to keep working with MASS in the future?  *Part c:*  Would you be willing to work with MASS in the future?   - Why - Needs |
| ***Closing***   - Is there anything I missed that I should know? - Questions interviewee - Explain summary of interview + ask for feedback - Thanks | |

**Students**

| ***Introduction:***   - Study- and interview purpose   - Specify: reasons for absence will not be discussed   - Give number from CYH physician in case student needs support after the interview - Anonymity, signing informed consent form - Permission for recording | |
| --- | --- |
| ***Topic*** | ***Questions*** |
| **Introduction interviewee** | Could please introduce yourself?   - Age |
| **Contact with school** | How did your mentor respond to your absence?   - When/how long after initial sick report - How - Satisfaction support from mentor   - Improvement points?   At what point were you invited for a conversation with your mentor about your absence?   - Satisfaction with conversation, usefulness |
| **Referral to CYH physician** | Were you referred to a CYH physician? *If no, continue to next topic*  When did your mentor propose a consultation with the CYH physician?   - Own thoughts about referral   What information did your mentor give you about the referral?   - Clarity, completeness - Did you understand why you were being referred? - Did you understand prior to the consultation what the purpose of the referral was? - Improvement points |
| **Consultation with CYH physician** | How much time was there between the conversation with your mentor and the consultation with the CYH physician?  How did you experience the consultation with the CYH physician?   - Satisfaction - Did you feel the CYH physician helped you? How?   What did you think about the action plan you and the CYH physician developed?   - Were you involved in the development of the plan?   - Satisfaction - Satisfaction with plan   Were your parents involved by the CYH physician?   - Satisfaction with parental involvement   Is there anything the CYH physician could have done better? |
| **After the consultation (not for students who were not referred)** | What happened after you visited the CYH physician?  To what extent did your action plan help you?   - Did you stick to the plan? - Did school stick to the plan?   To what extent did school support you after the consultation with the CYH physician?   - Satisfaction   Is there anything the school could have done better to help you? |
| **Satisfaction with MASS** | *check if they know the term MASS* What do you think about MASS?   - Did it help you? - Do you think it could help other students?   Is there anything about MASS that could be improved? |
| ***Closing***   - Is there anything I missed that I should know? - Questions interviewee - Explain summary of interview + ask for feedback - Thanks | |
